# Regression of a Fungating Tumor After Hypofractionated Radiation Therapy in a Patient With Metastatic Breast Cancer

**DOI:** 10.7759/cureus.1417

**Published:** 2017-07-02

**Authors:** Kevin Quackenbush, Arya Amini, Christine M Fisher, Rachel Rabinovitch

**Affiliations:** 1 Department of Radiation Oncology, University of Colorado School of Medicine

**Keywords:** breast cancer, palliative radiation, hypofractionated, primary tumor, metastatic breast cancer, locally advanced breast cancer

## Abstract

Radiation therapy is a well-established palliative treatment for symptomatic metastases from breast cancer. This is also true of symptomatic primary breast tumors in patients with metastatic disease or in those who are medically inoperable. Further, local progression in the chest wall can severely impair quality of life, with local pain, bleeding, and significant impact on one’s self-image. Here, we present the case of a patient who showed an exceptional response to a palliative hypofractionated radiation course to her bleeding, fungating breast primary.

## Introduction

Breast cancer is the most common cancer in women in the US and the second most common cause of cancer-related death in women. An estimated 252,710 new cases of breast cancer will be diagnosed in the US in 2017, with an estimated 40,610 deaths [1]. An estimated 5%-10% of breast cancer patients have distant metastatic disease at initial presentation, and current guidelines dictate that these patients should receive systemic treatment rather than therapies directed to their primary tumor [1-3]. A recent prospective randomized trial failed to demonstrate an improvement in the overall survival with locoregional treatment of the primary breast tumor in patients presenting with a metastatic disease [4]. However, palliative radiotherapy to the primary breast tumor in the metastatic setting can provide relief from a variety of symptoms including ulceration, bleeding, arm edema, pain, and brachial plexopathy [5].

## Case presentation

A 59-year-old woman presented in September 2014 with a left fungating breast tumor. She had been diagnosed two years prior with stage III, ER-/PR-/Her2+ G3 invasive ductal carcinoma and pursued alternative therapies in Mexico including tumor incision “to let the cancer out” as well as intravenous vitamin C therapy. The incision into her breast tumor never closed and she developed a bleeding, painful, malodorous fungating mass. Her staging computed tomography (CT) scan demonstrated metastases to the liver, bones, lungs, and pelvis. Repeat biopsy of the fungating mass revealed poorly differentiated ER+/PR-/Her2+ carcinoma. She was started on systemic treatment with docetaxel, trastuzumab, pertuzumab, and eventually letrozole. She was referred for palliative radiation to the left fungating breast tumor but deferred treatment. For the next two years, she continued systemic therapy and was eventually switched to ado-trastuzumab emtansine and letrozole. She was hospitalized on two separate occasions for acute pain and anemia related to repeat episodes of hemorrhage from her breast tumor. During those hospitalizations, she again deferred palliative radiation treatment. As she continued systemic treatment, her metastatic disease burden remained stable, but her fungating breast tumor had persistent progression.

**Figure 1:**
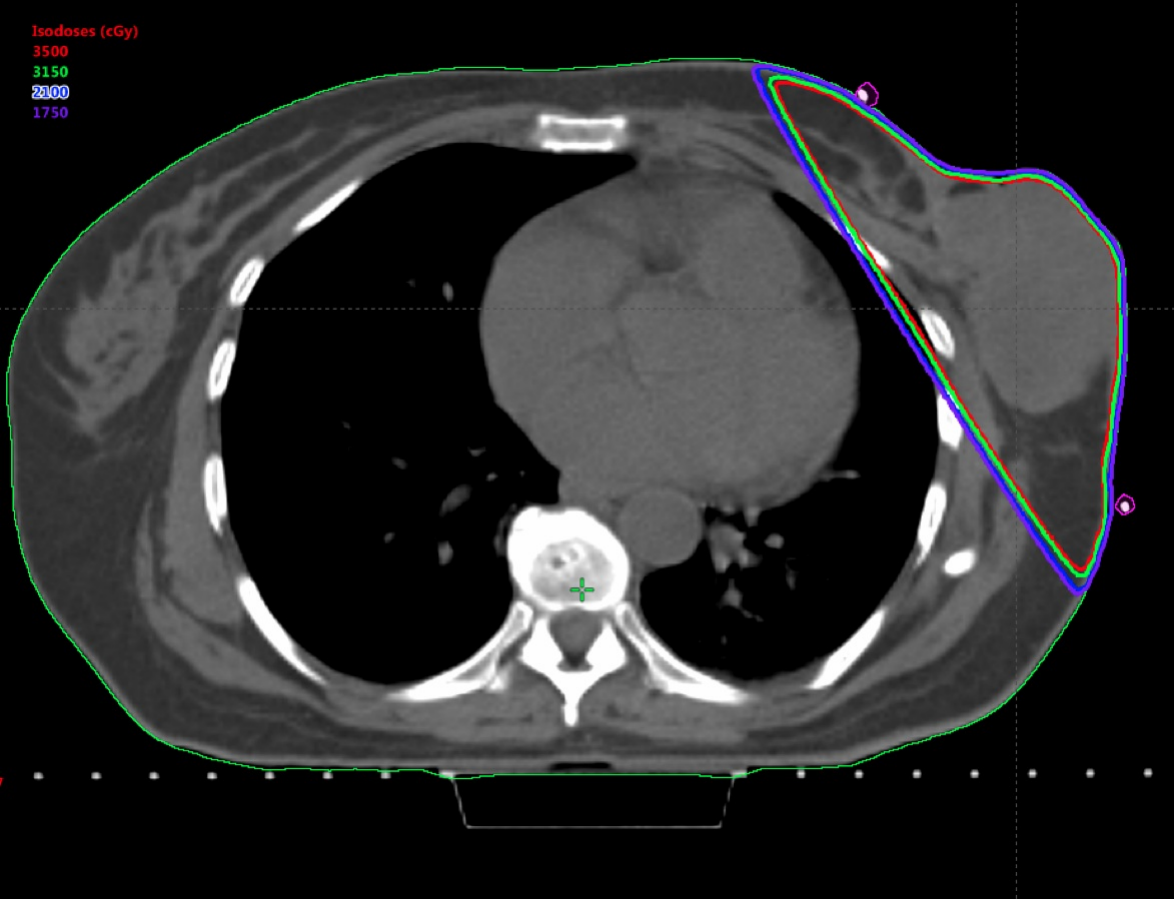
Axial image of the radiation treatment plan with representative isodose lines and corresponding doses (3500 cGy [red] prescription dose)

**Figure 2:**
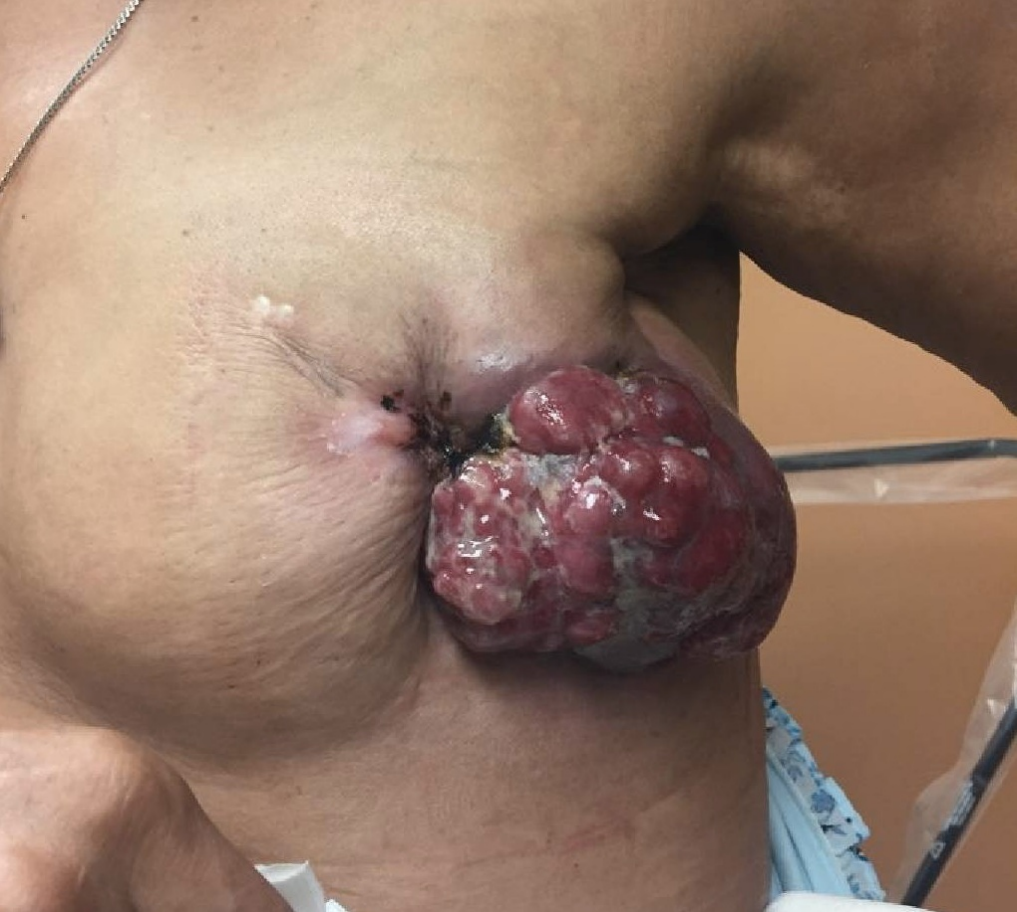
Pre-treatment left breast mass

**Figure 3:**
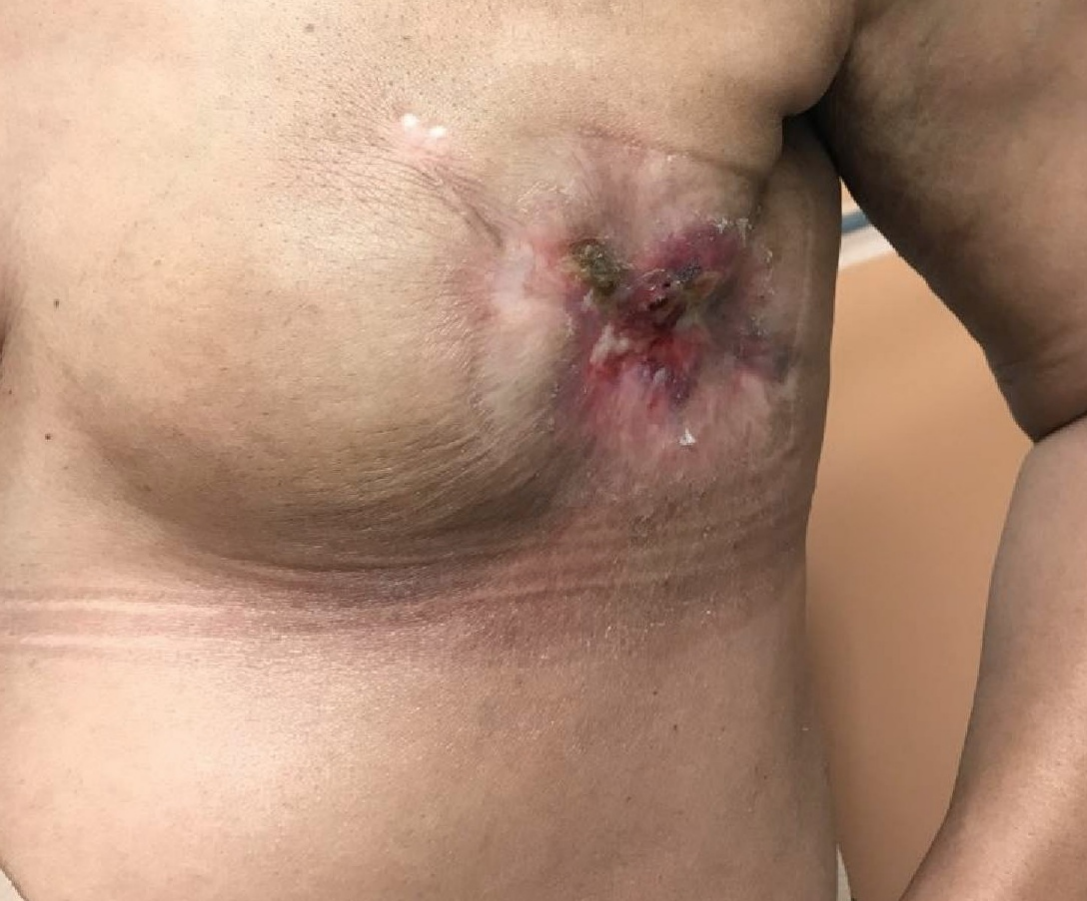
Six month post-treatment left breast mass

Due to this worsening local progression, she was referred back to radiation oncology for discussion of treatment. The symptoms stemming from her breast tumor included pain, odor, bleeding requiring repeat transfusions of red cells for blood loss, and emotional distress. Together these were significantly impairing her quality of life, and she opted for palliative radiation. At the time of treatment planning, the patient’s left breast tumor measured 10.9 cm in greatest dimension on the CT scan. A CT-based treatment plan was utilized (Figure 1). Tangential wedged photon fields were placed for planning. No bolus was applied. The setup included weekly electronic portal imaging and given the palliative nature of this treatment, no motion management was performed. She received 35 Gy in 5 fractions delivered every other day. Ado-trastuzumab emtansine and letrozole were continued during and after radiotherapy. The mean cardiac dose was 0.8 Gy and mean dose to the left lung was 0.9 Gy. She tolerated her accelerated treatment well and had some minimal tumor regression by the end of her treatment. Her only side effects from the treatment were grade 1 desquamation and grade 1 fatigue, both of which resolved. Over the next several months, her tumor continued to regress and she reported excellent improvement in pain, bleeding, and malodor. Her response continued with near complete regression of the tumor at six months post-treatment (Figures 2-3). 

## Discussion

There is an ongoing debate regarding the benefit of locoregional treatment of the breast with surgery and/or radiation in women whose initial breast cancer presentation includes distant metastatic disease. The question that remains to be answered is what effect locoregional treatment of the primary tumor may have on the overall survival in addition to other measures including patient-reported quality of life. There is retrospective data that has demonstrated a survival benefit to locoregional treatment, but there is concern that these studies have significant selection bias (i.e., younger women and/or women with better performance status may be more likely to be offered locoregional treatment) [3]. While this debate continues, the fact remains that many women with locally advanced or metastatic breast cancer, who have symptoms stemming from their primary breast tumor, can receive tremendous benefit from radiation therapy as a palliative treatment.

There is no guideline-based radiation dose for palliation of a primary breast tumor. Common palliative regimens such as 20 Gy in 5 fractions or 30 Gy in 10 fractions may be used in this setting. The benefit of 30-35 Gy in 5 fractions, which is a common regimen offered at our institution, is its higher biologic equivalent dose (BED) compared to other palliative regimens. This dose regimen yields a BED equivalent to 70 Gy in standard fractionation (2 Gy/fraction), which is a dose appropriate for gross disease in the setting of breast cancer. By comparison, a schedule of 30 Gy in 10 fractions delivers a BED of only 60 Gy. This, combined with the fact that a 35 Gy in 5 fraction schedule does not appear to significantly add more side effects and is more convenient for the patient, suggests that the treatment plan used in this case may be an ideal regimen for palliation of a locally advanced breast tumor.

## Conclusions

Palliative radiation therapy is an effective means of helping to control symptoms such as ulceration, bleeding, and malodor from locally advanced primary breast tumors in the metastatic setting. Longer follow-up is needed to establish the duration of the tumor and symptom control in this setting.
